# Assessing Impact of Data Quality in Early Post-Operative Glioblastoma Segmentation

**DOI:** 10.3390/jimaging12020073

**Published:** 2026-02-10

**Authors:** Ragnhild Holden Helland, David Bouget, Asgeir Store Jakola, Sébastien Muller, Ole Solheim, Ingerid Reinertsen

**Affiliations:** 1 Department of Health Research, SINTEF Digital, NO-7465 Trondheim, Norway; david.bouget@sintef.no (D.B.); sebastien.muller@sintef.no (S.M.); ingerid.reinertsen@sintef.no (I.R.); 2 Department of Circulation and Medical Imaging, Norwegian University of Science and Technology, NO-7491 Trondheim, Norway; 3 Department of Clinical Neuroscience, Institute of Neuroscience and Physiology, Sahlgrenska Academy, University of Gothenburg, 40530 Gothenburg, Sweden; jakola.asgeir@gu.se; 4 Department of Neurosurgery, Sahlgrenska University Hospital, 40530 Gothenburg, Sweden; 5 Department of Neurosurgery, St. Olavs Hospital, Trondheim University Hospital, NO-7030 Trondheim, Norway; ole.solheim@ntnu.no; 6 Department of Neuromedicine and Movement Science, Norwegian University of Science and Technology, NO-7491 Trondheim, Norway

**Keywords:** segmentation, glioblastoma, post-operative MRI, deep learning, data quality

## Abstract

Quantification of the residual tumor from early post-operative magnetic resonance imaging (MRI) is essential in follow-up and treatment planning for glioblastoma patients. Residual tumor segmentation from early post-operative MRI is particularly challenging compared to the closely related task of pre-operative segmentation, as the tumor lesions are small, fragmented, and easily confounded with noise in the resection cavity. Recently, several studies successfully trained deep learning models for early post-operative segmentation, yet with subpar performances compared to the analogous task pre-operatively. In this study, the impact of image and annotation quality on model training and performance in early post-operative glioblastoma segmentation was assessed. A dataset consisting of early post-operative MRI scans from 423 patients and two hospitals in Norway and Sweden was assembled, for which image and annotation qualities were evaluated by expert neurosurgeons. The Attention U-Net architecture was trained with five-fold cross-validation on different quality-based subsets of the dataset in order to evaluate the impact of training data quality on model performance. Including low-quality images in the training set did not deteriorate performance on high-quality images. However, models trained on exclusively high-quality images did not generalize to low-quality images. Models trained on exclusively high-quality annotations reached the same performance level as the models trained on the entire dataset, using only two-thirds of the dataset. Both image and annotation quality had a significant impact on model performance. In dataset curation, images should ideally be representative of the quality variations in the real-world clinical scenario, and efforts should be made to ensure exact ground truth annotations of high quality.

## 1. Introduction

Diffuse glioma is the most common malignant primary brain cancer, and glioblastoma is the most aggressive form, with a median survival of only 15 months post diagnosis [1]. Glioblastoma is usually treated by maximum safe surgical resection followed by radiation and chemotherapy [2], and the pre-operative, early post-operative, and follow-up magnetic resonance imaging (MRI) scans form the basis for treatment decisions. The extent of resection (EOR), defined as the ratio between the surgically removed and the pre-operative tumor volume, is one of the main prognostic factors for these patients [3]. Several openly available software tools exist for automatic or semi-automatic delineation of the pre-operative tumor volume [4,5,6]. Such methods are currently being implemented into clinical practice as part of modern neuro-navigation systems. The standard method for estimating the volume of the residual tumor from the early post-operative MRI (EPMR) scans has long been based on measurements prone to high inter-rater variability [7], such as the bi-dimensional product of the largest axial diameter of the contrast-enhancing residual tumor, according to the Response Assessment in Neuro-Oncology (RANO) criteria [8,9]. Exact automatic measurements of the pre- and post-operative tumor volumes based on automatic segmentation of the tumor in both the pre-operative and EPMR scans would allow for an automatic, efficient, objective and reproducible EOR calculation.

The emergence of fully convolutional neural networks has been a paradigm shift within the field of medical image segmentation. The U-Net architecture [10] has served as a baseline architecture for most of the state-of-the-art methods since it was introduced in 2015. The nnU-Net framework [11] represented a new important milestone in the field, with a method for optimizing the hyper-parameters and training configuration of the U-Net architecture. For the specific task of pre-operative glioblastoma segmentation, considerable advances have been made since the first MICCAI Brain Tumor Segmentation (BraTS) challenge in 2012 [12]. The winning submissions of the BraTS challenge since 2021 have all been modifications or ensembles based on the nnU-Net framework [13,14,15]. The winning contribution in 2023 introduced synthetic data in the training set [15], which increased the mean Dice score from 0.8821 in 2022 to 0.8853 in 2023 on the validation set. Such an incremental increase in segmentation performance clearly indicates a plateau has been reached, and a new gap will be difficult to bridge from a methodological standpoint alone without diversifying the dataset. As such, the pre-operative glioblastoma segmentation challenge was replaced by a post-treatment segmentation challenge in 2024 [16]. Additionally, the BraTS Glioma Segmentation in Sub-Saharan Africa Patient Population (BraTS-Africa) Challenge [17], featuring a more varied dataset in terms of image quality, represents a step in the right direction for the creation of robust and generic models. A shift in strategy can be clearly identified, highlighting the newfound interest in the research community to focus on generalization across MRI acquisition time points, scanners, geographical areas and image quality.

The dataset used in the BraTS post-treatment challenge was a combination of early post-operative images acquired immediately after surgery and follow-up images acquired several months later. Particular challenges related to post-operative segmentation, not featured in the pre-operative glioblastoma segmentation task, ought to be mentioned. In EPMR scans, small and fragmented lesions are mainly visible, leading to an extreme class imbalance, while follow-up scans often feature tumor regrowth. Furthermore, the presence of noise in and around the resection cavity complicates the annotation task. Enhancement caused by bleeding, tissue manipulation, infarctions, and hemostatic agents can easily be confounded with residual tumor tissue. Recent studies demonstrated the successful transfer of the current state-of-the-art models for pre-operative segmentation to the early post-operative use case [18,19,20,21,22], and established the clinical value of automatic EOR computation [23]. Although fairly close to the inter-rater variability of human expert raters on early post-operative segmentation [24], reported Dice scores of 50–60% for post-operative automatic segmentation are far from their pre-operative counterpart, reaching more than 90% [25]. Additionally, inter-rater variability studies conducted among expert annotators resulted in a large disagreement regarding the post-operative residual tumor to annotate, as compared to pre-operative tumor annotation [7,24].

Exact delineation of the residual tumor after surgery is not part of the standard clinical routine, and large variations in the quality of the EPMR scans can be witnessed between hospitals. In particular, the MRI acquisition type (i.e., volumetric or non-volumetric) and image resolution vary considerably. As stated in a recent overview study, data quality is a significant obstacle to the growth and adaptation of AI applications in clinical practice [26]. Several studies on similar medical image segmentation tasks have investigated the impact of quality on model performance through applying synthetic quality degradations, such as adding noise, contrast reduction, gamma correction, and simulated motion artifacts [27,28,29]. One study evaluated the relationship between MR image quality metrics (IQMs) and deep learning-based segmentation accuracy of brain tumors, suggesting that a significant relationship might exist between some specific MR IQMs and tumor segmentation model performance [30]. The effect of dataset size, image quality and image type on deep learning-based prostate segmentation from 3D ultrasound was assessed [31], where the image quality was found to have a significant effect on segmentation performance for one imaging direction. The quantitative impact of label noise on the quality of brain tumor segmentation from MRI scans has been investigated by simulating the biases and variances of different annotators with erosion and dilation of the dataset annotations [32]. It was shown that the performance of the segmentation models decayed as the scale of the label contamination increased in both directions. However, the performance decay was less significant with a random contamination of both erosion and dilation, simulating a mixture of different annotators. To summarize, all of these studies evaluated the model performance contrasted against the image and annotation quality of the test set, and a few of them investigated how training with different quality levels affected the performance [27,30,32]. Two of the found studies assessed the impact of annotation quality [27,32]. However, the annotation quality degradations were synthesized with data augmentation techniques, slightly limiting the implications to the actual quality variations seen in the clinic.

In this study, the impact of image and annotation quality on model training and performance in early post-operative glioblastoma segmentation was assessed. The Attention U-Net architecture, previously used for early post-operative glioblastoma segmentation [18], was selected for this purpose. The main contributions of the study are (i) dataset curation with expert assessment of image and annotation quality, (ii) model training with different quality-based subsets of the dataset, and (iii) validation studies and statistical analysis to assess the impact of data quality on trained models and segmentation performance.

## 2. Materials and Methods

### 2.1. Data

A dataset consisting of EPMR scans from 423 patients who underwent surgical resection of glioblastoma was used for this study. The dataset is a subset of a dataset used in a previous study [18], including data from 236 patients from Trondheim University Hospital (STO) in Trondheim, Norway, and 187 patients from Sahlgrenska University Hospital (SUH) in Gothenburg, Sweden.

All EPMR scans were acquired within the recommended time frame of 72 h after surgery, as stated by the National Comprehensive Cancer Network [33] guidelines, in order to maximize contrast differences between residual enhancing tumor and tissue enhancement due to post-surgical reparative changes in the tissue [34,35]. Two EPMR sequences were used, namely contrast-enhanced T1-weighted (T1-CE) and T1-weighted (T1w) MR scans. Details about average image resolution and spacing of the T1-CE sequences, for each of the two hospitals, can be found in Table 1.

The residual tumor tissue was manually annotated in 3D by expert neurosurgeons and neuroradiologists, using all available standard MR sequences. Residual tumor tissue was defined as enhancing tissue in the T1-CE sequence, appearing darker in the T1w scan, in order to eliminate enhancement caused by blood products after the surgery. Furthermore, a distinction was made between patients with a residual tumor (RT) volume larger than 0.175 mL [35], and those with a tumor volume below this threshold, considered as complete or gross total resections (GTR). The cut-off value was chosen to reduce the risk of misinterpretation when distinguishing between residual tumor enhancement and non-specific enhancement [35]. According to this definition, 274 patients (65%) in the dataset exhibited a residual tumor, and 149 patients (35%) had gross total resections.

### 2.2. Quality Evaluation Process

The data quality evaluation was performed by two expert neurosurgeons. One expert evaluated the quality of all images and annotations from both hospitals. The second expert evaluated only the data from SUH, in order to assess the inter-rater agreement of the quality evaluation. The quality of the images and annotations was determined based on one view from each volume in all three directions (i.e., axial, coronal, and sagittal). The views were selected based on the center of mass of the annotated tumor or the resection cavity. An example view for data quality evaluation is included in the Supplementary Material, Figure S1.

The image quality was rated on a scale from 1 to 3, signifying (1) high, (2) medium, and (3) low quality. Volumetric images with approximately isotropic voxel spacing of $1\;{\mathrm{mm}}^{3}$ and no visible artifacts were rated as high quality. The images were rated as medium or low quality depending on the grade of quality degradation, given the presence of artifacts such as aliasing due to movement in the MR scanner, or non-volumetric scans with high slice thickness. The quality of the annotations was rated on a qualitative scale with five levels: (1) perfect segmentation, (2) slightly undersegmented, (3) very undersegmented, (4) slightly oversegmented, and (5) very oversegmented.

Image quality metrics (IQMs) were calculated using the open-source toolbox MRQy [36], following a previous study on the impact of image quality on brain tumor segmentation performance [30]. The image and annotation qualities evaluated by the clinicians were compared against the quantitative IQMs from MRQy, and the slice thickness of the MRI scans, using correlation coefficients. This was done to assess the value of manually evaluated and qualitative image and annotation qualities, and place them within the context of previous related work. The distribution of MRI slice thicknesses for each quality category is presented in the Supplementary Material, Table S1. The correlation coefficients between the evaluated qualities, slice thickness, and IQMs from MRQy are also presented in the Supplementary Material, Figure S2, along with a more detailed description of the IQMs.

Following the initial analysis of the distribution of the evaluated qualities, both the image and annotation quality scales were simplified to two levels: high quality and low quality. As very few images fell within the “medium” quality category, and very few annotations were represented within the “very over- or under-segmented” categories, the sample sizes of these categories were too small to assess any statistically meaningful differences in segmentation model performance. Additionally, related studies have employed two quality levels [30], and the simplification to two levels ensured coherence and comparability with previous work. The images evaluated as medium and low quality were thus grouped together as low quality, and the annotations evaluated as slightly or very under- or oversegmented were grouped together as low quality.

The inter-rater agreement of the quality evaluation after simplification to two quality levels was performed solely based on the dataset from SUH, using contingency tables and Cohen’s kappa coefficient score.

### 2.3. Segmentation

Following previous work on pre- and post-operative glioblastoma segmentation [4,18,37], a patch-wise Attention U-Net architecture [38] was employed. Previous studies have shown that network architecture choices (i.e., patch-wise or full volume) and the number of included input sequences (i.e., FLAIR and pre-operative T1-CE) had an impact on voxel-wise segmentation performance and patient-wise RT vs. GTR classification performance [18]. A patch-wise architecture using the two main sequences for annotating residual tumor after glioblastoma surgery (i.e., T1w and T1-CE) was chosen, as the combination yielded the best voxel-wise segmentation performance. The Attention U-Net architecture was favored over nnU-Net given the proneness of the latter towards false positives, lowering the models’ patient-wise specificity considerably.

#### 2.3.1. Pre-Processing

The pre-processing of the images was kept to a minimum, with co-registration of the two sequences using the SyN diffeomorphic method from the Advanced Normalization Tools (ANTs) library [39], resampling to isotropic voxel size of $1\;{\mathrm{mm}}^{3}$, and tight cropping around the skull. In addition to the T1w and T1-CE sequences, a differential sequence of the two was added on the fly during training, as this was shown to improve the performance in the BraTS 2024 Adult Glioma Post-Treatment challenge [16]. Data loading and augmentation was handled by the MONAI library. As the two available sequences were co-registered, the differential sequence was simply computed by subtracting the T1w sequence from the T1-CE sequence, implemented as a custom data transform in the MONAI dataloader.

#### 2.3.2. Network Architecture and Training

The Attention U-Net [38] architecture was used with an input patch size of $160^{3}$ voxels, instance normalization, and five convolutional block levels, with {16, 64, 128, 256, 512} features on each of the levels, respectively. The network was pre-trained on the BraTS Adult Glioma Post-Treatment Challenge [16] dataset. The dataset consists of both early post-operative and follow-up MRI scans, with the same architecture and training parameters. The models were trained with a combination of cross-entropy and Dice loss, and the AdamW optimizer with an initial learning rate of $5\times 10^{-4}$. Gradient accumulation was employed with a batch size of 8 and 4 accumulated gradient steps, yielding an effective batch size of 32. Each model was trained for a maximum number of 500 epochs, with the best checkpoint selected based on the lowest validation loss monitored on the validation fold. During training, data augmentation techniques from the MONAI library, such as random cropping, flipping, intensity scaling, and affine transformations, were randomly selected and applied to the images. The model architecture, training, and validation pipeline were implemented in Python 3.11.9 using PyTorch 2.4.0, PyTorch Lightning v2.4.0, and MONAI v1.3.2.

#### 2.3.3. Post-Processing

After inference, the network predictions were resampled back to the original image space before removing predictions outside the brain to reduce noise. The resulting volumes were thresholded at 0.175 mL [35] for patient-wise classification of RT vs. GTR.

### 2.4. Quality Impact Experiments

To evaluate the impact of image and annotation quality on segmentation performances, the models were trained on different quality-based subsets of the dataset as described in the following:

- ImAnAll: The whole dataset. This was used as the reference model to compare the other models against.
- ImHigh: High-quality T1-CE images, irrespective of the annotation quality.
- AnHigh: High-quality annotations, irrespective of the image quality.
- ImAnHigh: Intersection of high-quality T1-CE images and high-quality annotations.

**Figure 1:**
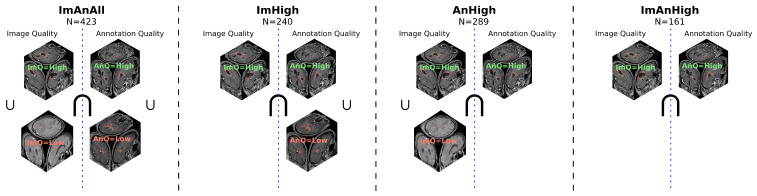
Overview of data quality inclusion criteria for each experiment.

A graphical representation of the training data inclusion criteria for each experiment is shown in Figure 1. More details about the training dataset for each experiment can be found in the Supplementary Material, Table S2.

### 2.5. Validation

All models were trained and validated using five-fold cross-validation with a two-way split. The dataset was split into five mutually exclusive folds, where each fold was used as a validation set in turn, while the model was trained on the remaining four folds. As such, each sample in the dataset was part of the validation set exactly once, and the validation scores for each experiment were computed for each of the five trained models over the respective validation set, effectively assessing the performance over the entire dataset for each experiment. Additionally, the folds were stratified according to image and annotation quality, ensuring equal representation of all data qualities within each fold. This allowed for computation of performance scores over the entire dataset for each of the four experiments, regardless of the quality criteria on the training set. The same folds were thus used for all experiments, filtered according to the respective quality criteria for each experiment, as illustrated in Figure 1. During validation, the original unfiltered folds were used, allowing for assessment of the model performance for all the quality categories, while ensuring no overlap between the training and validation sets.

#### 2.5.1. Metrics

The primary metrics used for evaluating the models’ voxel-wise segmentation performance were the Dice Similarity Coefficient (DSC, Dice score) and the 95% Hausdorff distance (HD95). These two metrics were calculated between the 3D volumes of the ground truth and predicted segmentations. The Dice scores are only reported over the “positive” samples, i.e., the patients exhibiting RT according to the ground truth annotation, as calculating Dice scores for images with empty segmentations does not yield any valuable information. Similarly, the HD95 distance metric is only defined for two non-empty segmentations, and thus only calculated for cases with both “positive” ground truth and “positive” prediction. In order to evaluate the models’ performances for the task of patient-wise classification of RT vs. GTR, recall/sensitivity, specificity, and balanced accuracy metrics were computed. Additional metrics, including the mean absolute volume differences, are reported in the Supplementary Material, Table S3.

#### 2.5.2. Statistics

A Bayesian mixed-effects linear model predicting Dice score was used to assess the difference between experiments, and quantify the effect of different image and annotation qualities in the validation set. The image and annotation qualities were the fixed effects. The experiment and patient label were random effects. The estimates and confidence intervals for fixed effects were calculated using the brms R package (v2.22.0) in R (v4.3.3), the posterior median $R^{2}$ and confidence interval were calculated with brms::bayes_R2, and the Leave-One-Out cross-validation-adjusted $R^{2}$ r2_loo was calculated using the performance package (v0.13.0). Dice scores were used to compare the intrinsic performance of the experiments. The resulting groups were tested using a pairwise Wilcoxon test for paired samples.

The Bonferroni correction of type I error was applied to compensate for multiple pairwise comparisons between groups. The effect sizes of the differences were assessed by Cohen’s $d=(\bar{x_{i}}-\bar{x_{j}})/s$, where $\bar{x_{i}}$ and $\bar{x_{j}}$ are the Dice scores achieved with models *i* and *j*, respectively, for paired samples. The pooled standard deviation *s* was calculated using clustered standard errors $S_{i}$ [40] from the regression. As all groups had the same number of observations *n*, this was reduced to $s^{2}=\left(S_{i}^{2}+S_{j}^{2}\right)/2$. Values of d=0.01, d=0.2 and d=0.5 were used for describing very small, small and medium effect sizes, respectively [41].

## 3. Results

### 3.1. Data Quality Evaluation

**Figure 2:**
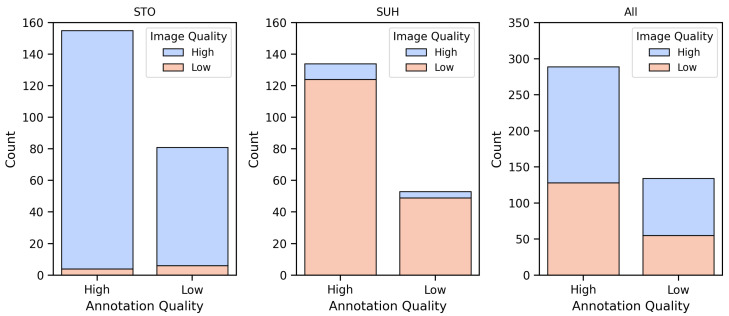
Distribution of evaluated image and annotation quality, from the two hospitals and overall.

The results of the quality evaluation performed by the first clinician are shown in Figure 2. The number of images and annotations within each category are shown as bar plots with one bar for each annotation quality level, and colors for contrasting image quality. The plot on the left shows the quality distribution for the data from STO, the middle one for SUH, and the right one for the entire dataset. The distributions are skewed towards one image quality for both hospitals, with mostly high-quality images for STO and mostly low-quality images from SUH. This was expected, as the images from SUH were mostly stacked 2D slices with high slice thicknesses (approximately 5 mm), which was one of the main criteria for low image quality. The distribution of the annotation qualities is skewed towards high quality, with about one-third of the annotations in the low-quality category.

To assess the validity of the quality evaluation metrics used in this study compared to previous related studies [30], IQMs were calculated using MRQy [36]. Correlation coefficients were calculated between the evaluated image and annotation qualities, the IQMs, and MRI slice thickness. These are presented in the Supplementary Material, Figure S2, along with a more detailed explanation of the IQMs and their relation to image quality. The analysis of the correlation coefficients revealed some shortcomings of the IQMs in their ability to identify scans with low resolution in the sagittal and coronal planes. As each IQM was calculated per 2D slice in the axial direction, the metrics failed to capture low visual quality due to non-volumetric (2.5D) acquisitions and high slice thickness. The 2.5D scans with 5 mm slice thickness were often rated as higher image quality according to some of the IQMs than the high-resolution 3D acquisitions. The manually evaluated image qualities were strongly correlated with slice thickness. This was expected, as slice thickness was the main criterion for evaluating low image quality, in addition to the presence of artifacts such as aliasing due to movement in the scanner.

The data originating from SUH was evaluated by a second neurosurgeon to assess the inter-rater agreement of the quality evaluation. The counts of the evaluated image and annotation qualities by each of the evaluators are shown as contingency tables in Table 2, with the first observer’s evaluated qualities along the rows and the second observer’s evaluated qualities along the columns. The image and annotation qualities are shown in the left and right tables, respectively. According to the image quality table, the two clinicians agreed on nearly all the images evaluated in both the high- and low-quality categories, with the exception of one, which resulted in a Cohen’s kappa score of 0.96. The two observers had a lower agreement on the annotation quality. Most of the annotations fell within the high-quality category for both; however, they disagreed on a total of 54 cases and reached a Cohen’s kappa score of 0.23. The low agreement indicates subjectivity in assessing the quality of the post-operative residual tumor delineations, and reflects the inherent ambiguity of the post-operative segmentation task.

### 3.2. Experiment Results

The voxel-wise segmentation and patient-wise classification results for all experiments, evaluated on the entire dataset, are reported in Table 3. Segmentation performance metrics are reported for positive cases (patients exhibiting a residual tumor according to the ground truth annotation), as Dice and HD95 are uninformative for empty ground truth segmentations. Overall, the highest scores were obtained in the first experiment trained on all available data, ImAnAll, both for the voxel-wise segmentation performance and patient-wise classification performance. Most remarkable was the performance achieved in experiment AnHigh trained on exclusively high-quality annotations, employing two-thirds of the full dataset and still achieving a Dice score of 59.75% (1.3% below the top score), and a balanced accuracy of 60.41% (0.13% below the top score). Regarding the patient-wise classification performance of RT vs. GTR, the sensitivity was generally high, but specificity remained low across all experiments, indicating a tendency towards over-prediction of residual tumor.

The statistical analysis showed that the image quality and annotation quality of the validation data had a significant influence on the Dice scores. The reduction in Dice per increment of quality score was −0.127 [−0.179, −0.074] and −0.069 [−0.123, −0.014] for image and annotation quality, respectively, reported as mean and confidence interval. The regression achieved an $R^{2}=0.746$ [0.727, 0.762] and a LOO-adjusted $R^{2}=0.675$ [0.629, 0.714], meaning that 75% of the variance is explained by the regression model (respectively 68% LOO-adjusted), which is regularly considered a strong fit. The results from the pairwise Wilcoxon test between experiments for estimated Dice at marginalized fixed effects, reported in Table 4, showed a significant difference from ImAnAll to ImHigh and ImAnHigh ($p<10^{-11}$), and to AnHigh (p=0.020). However, the effect size of the difference between ImAnAll and AnHigh was very small (Cohen’s d=0.089), making the two practically indistinguishable.

**Figure 3:**
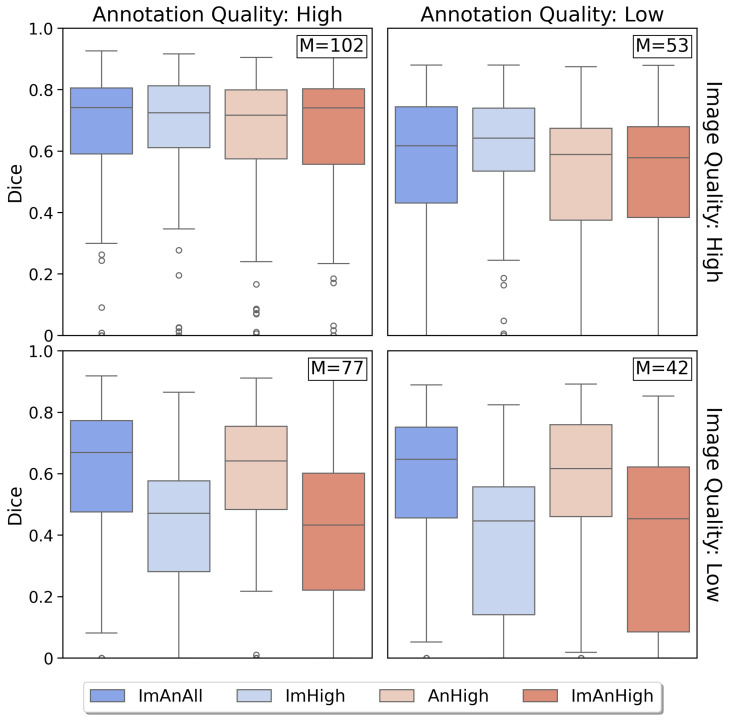
Dice score distributions stratified by image and annotation quality. The image qualities are displayed along the rows, annotation qualities along the columns, experiments are coded by color, and the number of patients within each quality level is shown as “M”. Models trained without low-quality images (ImHigh, ImAnHigh) generalize poorly to low-quality test images, whereas models trained on diverse image quality (ImAnAll, AnHigh) maintain performance.

To evaluate how training with different data quality restrictions affects the performance, boxplots of the Dice scores for each experiment contrasted according to image and annotation quality are displayed in Figure 3. Each row corresponds to an image quality from high to low, each column corresponds to an annotation quality from high to low, and the Dice scores between 0 and 1 are shown on the y-axis. The distribution of the Dice scores of images with both high image and annotation quality are remarkably similar across experiments, which may indicate that adding more data of diverse qualities does not deteriorate the results on the high-quality data. The scores for the high-quality images seem to decline steadily for all experiments with lower-quality annotations, although the scores are quite evenly distributed among experiments within each annotation quality level. This is to be expected, as both over- and undersegmented ground truths should have a lower agreement with the predictions than a perfect ground truth segmentation, if the predictions are correct. For the low-quality images, it can be observed that the Dice scores from the experiments where these were excluded from the training set (ImHigh and ImAnHigh) were considerably lower than for the two top-performing experiments (ImAnAll and AnHigh), with a similar trend for both annotation quality levels.

**Figure 4:**
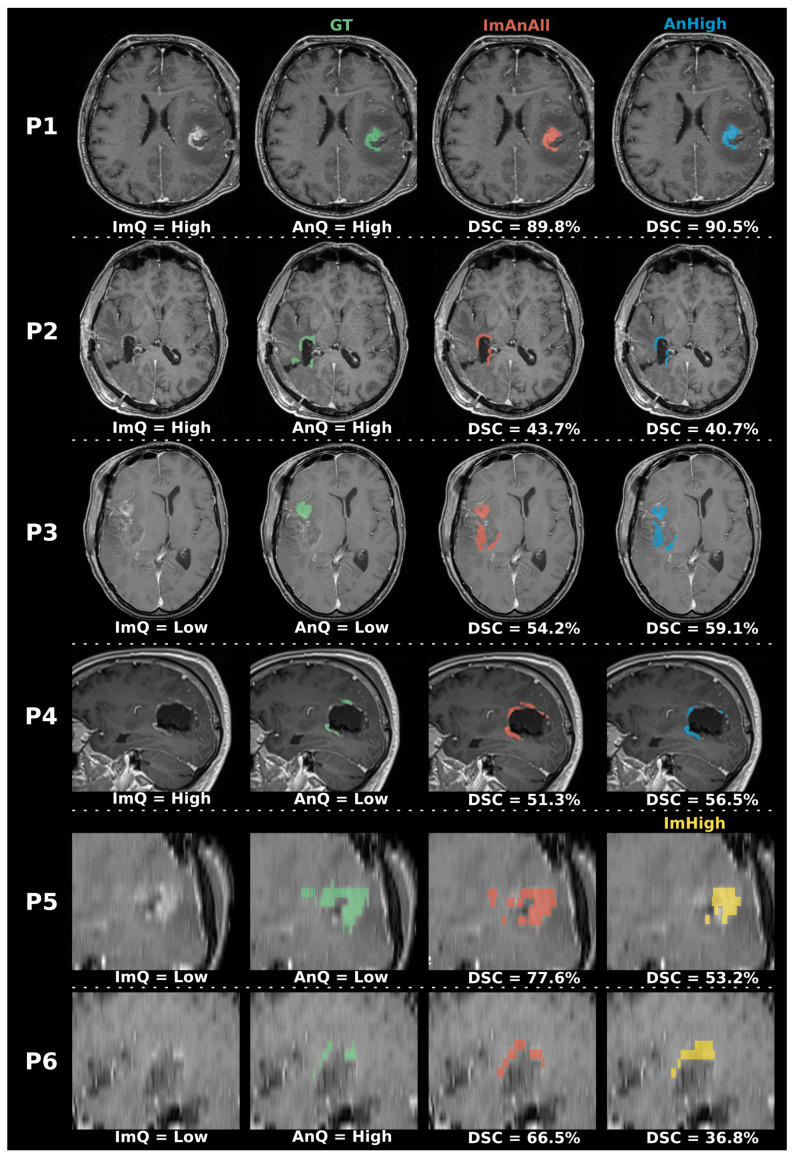
Example results for 6 cases (P1–P6). From the left: raw T1-CE EPMR scan, ground truth overlay (GT), ImAnAll experiment model prediction, AnHigh/ImHigh experiment model prediction. The first two cases, P1 and P2, illustrates the similar performance of the reference experiment ImAnAll and AnHigh. The third case P3 is an example of a low-quality image where the annotation quality was evaluated as low (very undersegmented), and where the models from ImAnAll and AnHigh detected a larger residual tumor than the annotator. Similarly, P4 shows an example of a high-quality image where the annotation was evaluated as low quality (slightly undersegmented), and where the models from ImAnAll and AnHigh detected a larger residual tumor in the vicinity of the resection cavity. Both P3 and P4 are examples that illustrate how the models in some cases might detect residual tumor that was missed by the expert annotator. Finally, P5 and P6 illustrate the poor performance of the model trained on exclusively high-quality images ImHigh on low-quality images. In P5, the model from ImHigh fails to detect a large part of the residual tumor, whereas in P6, the tumor is oversegmented compared to the ground truth and ImAnAll.

Example predictions from three models are shown in Figure 4, with interpretations in the caption.

## 4. Discussion

In this study, the impact of image and annotation quality on model training and performance in early post-operative glioblastoma segmentation was assessed. It was shown that models trained on exclusively high-quality images segmented images of low quality to produce Dice scores significantly lower than those produced from models trained on all data, meaning that models trained on exclusively high-quality images did not generalize well to low-quality images. Including images of lower quality in the training set, however, did not deteriorate model performance on high-quality images. Regarding the annotation quality, including low-quality annotations in the training dataset did not impact the model performance on high-quality annotations and images. However, the performance of the models trained with exclusively high-quality annotations, using two-thirds of the entire dataset, was equivalent to the performance of the models trained with more data and lower annotation qualities. The statistical analysis revealed that both image and annotation quality had a significant influence on the Dice scores.

### 4.1. Data Quality Impact on Segmentation Performance

As shown in Figure 3, all experiments achieved similar segmentation performances on patients with high-quality images and high-quality annotations, regardless of the quality of the training data. Removing images of low quality from the training data did not improve the model performance on the high-quality data. Conversely, adding more data of lower quality to the training set did not deteriorate the performance of the model on high-quality data. However, the models trained on exclusively high-quality images (ImHigh and ImAnHigh) did not generalize well to low-quality images, and the performances were significantly worse on low-quality images than the performance of the baseline model ImAnAll. These observations are in line with the conclusions from similar previous studies on data quality, stating that the training set should preferably contain images of various qualities in order to generalize to data of lower quality [26].

For the annotation qualities, the overall performance of each experiment seemed to decline with decreasing quality levels, as shown in Figure 3, with a most visible effect for the high-quality images. Although the performance of the model trained with exclusively high-quality annotations AnHigh was significantly different from the reference model ImAnAll, the effect size of the difference was very small. This means that the Dice scores predicted by either of them do not differ from those by the other in a practically meaningful way, although the training sample size of AnHigh was only two thirds of the full dataset. This indicates that the low-quality annotations do not add more valuable information to the training. Granted similar segmentation performance can be achieved using less data and higher quality annotations, more effort and emphasis should be dedicated to the annotation process, potentially at the expense of data quantity.

### 4.2. Classification Performance and Clinical Usability

Consistently low specificity was observed across all experiments for the patient-wise classification of RT and GTR, with values ranging from 13 to 26%. Although the sensitivity was high for all experiments, ranging from 90 to 96%, this reveals a tendency to over-predict residual tumor tissue. From a clinical point of view, a high model sensitivity could be acceptable, as it could potentially increase the sensitivity of the clinician assessing the post-operative images. On the other hand, a low specificity could limit the models’ applicability in prognostic stratification, for example, if the models were employed for automatic residual tumor quantification in clinical trials. There are several factors that might have contributed to this sensitivity-specificity imbalance. First, the threshold for distinguishing RT from GTR at 0.175 is sensitive to small segmentation errors, and even minor over-segmented areas could count as false positives. Additionally, the noise usually present in the surgical cavity, such as enhancement from blood products, in particular in low-resolution or high slice thickness scans, is often confounded with residual tumor even by expert annotators and can thus easily be misinterpreted by the models. Furthermore, the low inter-agreement for annotation quality evaluation gives an indication of subjective annotations, which means that model detections might reflect true residual tumor enhancement that was under-segmented by the human annotator.

The cases P3 and P4 in Figure 4 are examples of cases where the manual annotations have been labeled as very and slightly under-segmented, respectively. The two top-performing models detected larger residual tumors than the ground truth annotations in both cases, illustrating how automatic models can be useful in detecting possible residual tumors that might have been misinterpreted by the clinician. The automatic segmentation models could thus potentially increase the sensitivity of the clinician in the identification of residual tumor tissue, if used as an assistive tool in the clinic. Additionally, the overall high performance of all models on the high-quality images should be a strong motivation for clinicians to collect high-quality volumetric images for follow-up of glioblastoma patients.

### 4.3. Comparison with Previous Work and IQMs

Compared to previous studies, the perceived visual image and annotation qualities’ impact on model performance was investigated in this work. Most of the related previous studies on quality impact employed IQMs for image quality evaluation. To assess the validity of the quality metrics used in this study, IQMs were calculated with MRQy [36] and correlated with the quality metrics evaluated by expert clinicians. The analysis of the correlation coefficients revealed some shortcomings of the IQMs in their ability to identify scans with low resolution in the sagittal and coronal planes. The manually evaluated image qualities were strongly correlated with slice thickness, as this was the main criterion for evaluating low image quality, in addition to the presence of artifacts such as aliasing due to movement in the scanner. However, both the manually evaluated image quality and slice thickness had a low or negative correlation with several of the IQMs, meaning that some of the IQMs indicated higher quality for low-resolution scans.

Although the method for evaluating image and annotation quality in this study was qualitative and prone to subjectivity, the criteria for assessing the quality were clearly defined, and the inter-rater agreement of the image quality assessment was high. The visual quality and level of detail in the images are clearly dependent on the image resolution and the presence of artifacts, which the aforementioned quantitative IQMs used in related studies failed to capture. The manually assessed image and annotation qualities and their impact on model performance should therefore be of interest, and this study adds a new perspective to the existing literature on data quality impact in brain tumor segmentation. In addition, most of the studies trained the model once on the entire dataset and evaluated the performance according to different quality levels. Only a few of the found studies assessed the effect of training with different qualities [27,30,32]. Two studies assessed the impact of the annotation quality, both of them employing data augmentation techniques to add artificial noise to the ground truth segmentations [27,30]. As such, this study is most likely one of very few to investigate how the actual quality of the annotations as evaluated by a second clinician impacts the model, as well as the impact of visual image quality related to slice thickness and presence of artifacts.

### 4.4. Inter-Rater Agreement and Practical Recommendations

The motivation for this study was to investigate the impact of image and annotation quality on model performance in early post-operative segmentation to contribute to improved image analysis and interpretation in the clinical context of glioblastoma treatment. The post-operative glioblastoma segmentation task is known to pose some particular challenges compared to the analogous pre-operative segmentation task, with high inter-rater variability [24], and subpar segmentation performances for both expert raters and models [18]. Manual annotations by one or several expert clinicians are usually employed as ground truth segmentations when training deep learning models for medical image segmentation. The low inter-rater agreement of the annotation quality evaluation, in addition to the declining model performance with declining annotation quality, demonstrates the subjective nature of the manual annotations. Low inter-rater agreement on annotation quality might imply that part of the observed performance degradation on low-quality annotations may reflect label noise rather than model failure. This should be taken into consideration when interpreting voxel-wise metrics such as the Dice score, which measures the agreement with a subjective reference, rather than reflecting the absolute accuracy of the segmentation.

Recent years have seen a rise in medical image analysis through federated learning [42,43]. As opposed to collecting a large dataset in one place for training, a model is iteratively trained from center to center, each holding private their local dataset. As such, concrete guidelines and best practice advice for data collection and annotation are needed in order for each center to perform the task optimally. A common practice in dataset curation, particularly for competitions and challenges, is to remove low-quality data, as excluding non-volumetric, low-resolution, or noisy scans tends to incidentally boost model performance. This was confirmed by the results in this study, where high performances were achieved by all models on high-quality images and annotations, irrespective of the quality of the training data. As highlighted by a recent study [26], eliminating non-volumetric and low-quality images from clinical practice would probably have the greatest impact on model performance. However, if a dataset does not reflect the quality of the data seen in the real-world clinical setting, the reported performance will be artificially high. To summarize, image diversity and annotation quality should be the two prime criteria to consider for quality in dataset curation when attempting to build a dataset for glioblastoma segmentation.

### 4.5. Limitations

All experiments were conducted using a fixed Attention U-Net configuration to isolate the effect of data quality from architectural variability. Although absolute performance may differ with other architectures, the observed relative trends across data quality conditions are expected to generalize to other convolutional encoder–decoder models for early post-operative segmentation.

The image and annotation qualities were evaluated manually by an expert neurosurgeon, based on one axial, one coronal, and one sagittal view from each MRI volume. The evaluated qualities were thus biased towards the clinician who performed the evaluation. The preliminary inter-rater evaluator agreement study revealed an image quality bias linked to the annotator’s daily practice rather than any intrinsic quality. More precisely, the two clinicians often disagreed in the medium and low image quality categories, which were later merged into one category for low quality. A clinician working solely on volumetric MR scans will consider non-volumetric scans to be of low quality, while a clinician using mostly non-volumetric MR scans in clinical practice will have a more lenient quality assessment. One plausible explanation for the disagreement is then the vague definition of the evaluation criteria for separating medium from low-quality images. However, the clinicians had a near-perfect agreement in the high-quality image category. The distribution of each of the clinicians’ evaluated annotation qualities were quite similar, although there was some disagreement in the medium quality category. This highlights the subjective nature of the ground truth annotations, as both expert clinicians sometimes disagree with the annotator about the location and size of the residual tumor, and among themselves on the quality of previous annotations.

The effect of the sample size N on the segmentation performance could possibly interfere with the effect of the quality of the data without being captured by the statistical model. Bootstrapping of the training sets with stratified sampling of the qualities, to ensure equal training sample sizes, could possibly have eliminated this effect. However, the DSC boxplots in Figure 3 for the group showcasing both high image and annotation quality (top left corner) show similar performances of all experiments, indicating that a lower training sample size N does not have an effect on the performance within one quality level. Additionally, the AnHigh experiment achieved a performance equivalent to the reference experiment ImAnAll trained on all available data, with a considerably smaller training sample size. The size differences were thus deemed not to have a strong impact on the scores. Additionally, other works have studied the impact of the training sample size on the model performance [31], and as the purpose here was to study the impact of quality, it was deemed more pertinent to make use of the entire dataset in the training.

The dataset used in this study originated from two Nordic centers with specific acquisition protocols, particularly regarding slice thickness and volumetric coverage. While this enabled controlled analysis of quality impact, the findings may not generalize to other regions, scanner vendors, or clinical workflows. Further external or multi-regional validation will be necessary to confirm broader generalizability. Ideally, the number of samples within each quality category should be more evenly distributed. In future work, more data from other regions and hospitals should be included and evaluated to ensure better representation of all quality levels. Additionally, image quality degradations could be added to both images and annotations by data-augmentation techniques such as noise addition and downsampling, to increase the sample sizes in the under-represented quality groups.

## 5. Conclusions

An assessment of the impact of image and annotation quality on model performance in early post-operative glioblastoma segmentation was presented in this study. Both image and annotation quality were shown to have a significant impact on model performance. In dataset curation, images should ideally be representative of the quality variations in the real-world clinical scenario, and efforts should be made to ensure exact ground truth annotations of high quality. Manual assessment of early post-operative images is a difficult task with high inter-rater variability, and an automatic segmentation method could potentially increase the sensitivity of the clinician evaluating the images.

## Figures and Tables

**Table 1:** Dataset statistics grouped by hospital: number of patients, number of patients exhibiting residual tumor (RT), average resolution and average spacing of the T1-CE sequences. Resolution and spacing are reported as H × W × D.

Hospital	Patients	RT	Res. T1-CE [voxels]	Spac. T1-CE [mm]
STO	236	161 (68%)	321 × 387 × 228	0.81 × 0.74 × 1.05
SUH	187	113 (60%)	717 × 717 × 46	0.35 × 0.35 × 5.08

**Table 2:** Contingency tables of inter-rater agreement on the image and annotation quality of the SUH dataset.

Image Quality				Annotation Quality			
		Rater 2				Rater 2	
Rater 1		High	Low	Rater 1		High	Low
	High	13	1		High	110	23
	Low	0	173		Low	31	20

**Table 3:** Voxel-wise segmentation performance and patient-wise classification performance for all experiments. All metrics are reported as mean ± std, and all metrics are reported in % except the HD95 distance, reported in mm. For the segmentation performance metrics, the mean and std were calculated on a patient level for positive cases, i.e., patients exhibiting a residual tumor, whereas the mean and std of the classification metrics were calculated between folds.

Model	Segmentation		Classification		
	DSC	HD95 (mm)	Sens.	Spec.	bAcc
ImAnAll	61.05 ± 23.56	11.35 ± 13.89	94.96 ± 3.17	26.33 ± 14.46	60.65 ± 7.09
ImHigh	52.99 ± 26.22	24.75 ± 28.00	91.83 ± 2.73	13.34 ± 8.38	52.59 ± 2.98
AnHigh	59.75 ± 23.12	11.83 ± 13.53	96.81 ± 2.19	24.02 ± 6.02	60.42 ± 3.05
ImAnHigh	51.90 ± 26.97	23.53 ± 27.49	90.96 ± 2.85	18.20 ± 5.59	54.58 ± 1.63

**Table 4:** Effect sizes and *p*-values from pairwise comparison of the Dice scores from each experiment. The Bonferroni-corrected *p*-values are shown in the upper right part of the table on a red background, where *p* < 0.05 indicates statistical significance. The effect sizes of the differences between the experiments are quantified by Cohen’s *d*, shown on the bottom left side of the table on a blue background. Values of *d* ∼ 0.01, *d* ∼ 0.2 and *d* ∼ 0.5 indicate very small, small, and medium effect size, respectively.

	ImAnAll	ImHigh	AnHigh	ImAnHigh
ImAnAll	-	<$10^{-11}$	0.020	<$10^{-13}$
ImHigh	0.426	-	<$10^{-5}$	1.000
AnHigh	0.089	−0.346	-	<$10^{-7}$
ImAnHigh	0.456	0.069	0.403	-

## Data Availability

The dataset analyzed in this study is available from the corresponding author on reasonable request. The source code for model validation and metrics computation can be found at https://github.com/dbouget/validation_metrics_computation (accessed on 21 January 2026).

## Supplementary Materials

The following supporting information can be downloaded at https://www.mdpi.com/article/10.3390/jimaging12020073/s1, Figure S1: Example slide shown to a clinician for evaluation of image and annotation quality for one patient; Table S1: Minimum, mean, and maximum slice thickness (ST) for each quality category; Figure S2: correlation coefficients between the evaluated qualities, slice thickness, and IQMs from MRQy; Table S2: Number of images available for training, number of positive images and positive ratio, and patients from each hospital, for each experiment; Table S3: Additional metrics: Mean absolute volume difference ± std between prediction and ground truth in ml (VD), Sum of predicted positives (PP), true positives (TP), false positives (FP), true negatives (TN), and false negatives (FN) over 5 folds per experiment. Supplementary material references: [30,36].

## References

[1] Grochans S., Cybulska A.M., Simińska D., Korbecki J., Kojder K., Chlubek D., Baranowska-Bosiacka I. (2022). Epidemiology of glioblastoma multiforme–literature review. Cancers.

[2] Davis M.E. (2016). Glioblastoma: Overview of disease and treatment. Clin. J. Oncol. Nurs..

[3] Coburger J., Wirtz C.R., König R.W. (2017). Impact of extent of resection and recurrent surgery on clinical outcome and overall survival in a consecutive series of 170 patients for glioblastoma in intraoperative high field magnetic resonance imaging. J. Neurosurg. Sci..

[4] Bouget D., Alsinan D., Gaitan V., Helland R.H., Pedersen A., Solheim O., Reinertsen I. (2023). Raidionics: An open software for pre- and postoperative central nervous system tumor segmentation and standardized reporting. Sci. Rep..

[5] Huber T., Alber G., Bette S., Boeckh-Behrens T., Gempt J., Ringel F., Alberts E., Zimmer C., Bauer J.S. (2017). Reliability of Semi-Automated Segmentations in Glioblastoma. Clin. Neuroradiol..

[6] Vezhnevets V., Konouchine V. (2005). GrowCut: Interactive multi-label ND image segmentation by cellular automata. Proc. Graph..

[7] Berntsen E.M., Stensjøen A.L., Langlo M.S., Simonsen S.Q., Christensen P., Moholdt V.A., Solheim O. (2020). Volumetric segmentation of glioblastoma progression compared to bidimensional products and clinical radiological reports. Acta Neurochir..

[8] Wen P.Y., Macdonald D.R., Reardon D.A., Cloughesy T.F., Sorensen A.G., Galanis E., DeGroot J., Wick W., Gilbert M.R., Lassman A.B. (2010). Updated response assessment criteria for high-grade gliomas: Response assessment in neuro-oncology working group. J. Clin. Oncol..

[9] Wen P.Y., van den Bent M., Youssef G., Cloughesy T.F., Ellingson B.M., Weller M., Galanis E., Barboriak D.P., de Groot J., Gilbert M.R. (2023). RANO 2.0: Update to the Response Assessment in Neuro-Oncology Criteria for High- and Low-Grade Gliomas in Adults. J. Clin. Oncol..

[10] Ronneberger O., Fischer P., Brox T. (2015). U-net: Convolutional networks for biomedical image segmentation. International Conference on Medical Image Computing and Computer-Assisted Intervention.

[11] Isensee F., Jäger P.F., Full P.M., Vollmuth P., Maier-Hein K.H. (2021). nnU-Net for Brain Tumor Segmentation. International MICCAI Brainlesion Workshop.

[12] Menze B.H., Jakab A., Bauer S., Kalpathy-Cramer J., Farahani K., Kirby J., Burren Y., Porz N., Slotboom J., Wiest R. (2015). The Multimodal Brain Tumor Image Segmentation Benchmark (BRATS). IEEE Trans. Med. Imaging.

[13] Luu H.M., Park S.H. (2021). Extending nn-UNet for brain tumor segmentation. International MICCAI Brainlesion Workshop.

[14] Zeineldin R.A., Karar M.E., Burgert O., Mathis-Ullrich F. (2022). Multimodal CNN Networks for Brain Tumor Segmentation in MRI: A BraTS 2022 Challenge Solution. International MICCAI Brainlesion Workshop.

[15] Ferreira A., Solak N., Li J., Dammann P., Kleesiek J., Alves V., Egger J. (2024). How we won BraTS 2023 Adult Glioma challenge? Just faking it! Enhanced Synthetic Data Augmentation and Model Ensemble for brain tumour segmentation. arXiv.

[16] de Verdier M.C., Saluja R., Gagnon L., LaBella D., Baid U., Tahon N.H., Foltyn-Dumitru M., Zhang J., Alafif M., Baig S. (2024). The 2024 Brain Tumor Segmentation (BraTS) Challenge: Glioma Segmentation on Post-treatment MRI. arXiv.

[17] Adewole M., Rudie J.D., Gbadamosi A., Toyobo O., Raymond C., Zhang D., Omidiji O., Akinola R., Suwaid M.A., Emegoakor A. (2023). The Brain Tumor Segmentation (BraTS) Challenge 2023: Glioma Segmentation in Sub-Saharan Africa Patient Population (BraTS-Africa). arXiv.

[18] Helland R.H., Ferles A., Pedersen A., Kommers I., Ardon H., Barkhof F., Bello L., Berger M.S., Dunås T., Nibali M.C. (2023). Segmentation of glioblastomas in early post-operative multi-modal MRI with deep neural networks. Sci. Rep..

[19] Luque L., Skogen K., MacIntosh B.J., Emblem K.E., Larsson C., Bouget D., Helland R.H., Reinertsen I., Solheim O., Schellhorn T. (2024). Standardized evaluation of the extent of resection in glioblastoma with automated early post-operative segmentation. Front. Radiol..

[20] Bianconi A., Rossi L.F., Bonada M., Zeppa P., Nico E., De Marco R., Lacroce P., Cofano F., Bruno F., Morana G. (2023). Deep learning-based algorithm for postoperative glioblastoma MRI segmentation: A promising new tool for tumor burden assessment. Brain Inform..

[21] Cepeda S., Romero R., García-Pérez D., Blasco G., Luppino L.T., Kuttner S., Arrese I., Solheim O., Karlberg A., Pérez-Núñez A. (2024). Postoperative glioblastoma segmentation: Development of a fully automated pipeline using deep convolutional neural networks and comparison with currently available models. arXiv.

[22] Ghaffari M., Samarasinghe G., Jameson M., Aly F., Holloway L., Chlap P., Koh E.S., Sowmya A., Oliver R. (2022). Automated post-operative brain tumour segmentation: A deep learning model based on transfer learning from pre-operative images. Magn. Reson. Imaging.

[23] Majewska P., Holden Helland R., Ferles A., Pedersen A., Kommers I., Ardon H., Barkhof F., Bello L., Berger M.S., Dunås T. (2025). Prognostic value of manual versus automatic methods for assessing extents of resection and residual tumor volume in glioblastoma. J. Neurosurg..

[24] Visser M., Müller D.M., van Duijn R.J., Smits M., Verburg N., Hendriks E.J., Nabuurs R.J., Bot J.C., Eijgelaar R.S., Witte M. (2019). Inter-rater agreement in glioma segmentations on longitudinal MRI. Neuroimage Clin..

[25] Liu Z., Tong L., Chen L., Jiang Z., Zhou F., Zhang Q., Zhang X., Jin Y., Zhou H. (2023). Deep learning based brain tumor segmentation: A survey. Complex Intell. Syst..

[26] Bonada M., Rossi L.F., Carone G., Panico F., Cofano F., Fiaschi P., Garbossa D., Di Meco F., Bianconi A. (2024). Deep Learning for MRI Segmentation and Molecular Subtyping in Glioblastoma: Critical Aspects from an Emerging Field. Biomedicines.

[27] Güneş A.M., van Rooij W., Gulshad S., Slotman B., Dahele M., Verbakel W. (2023). Impact of imperfection in medical imaging data on deep learning-based segmentation performance: An experimental study using synthesized data. Med. Phys..

[28] Kugelman J., Alonso-Caneiro D., Read S.A., Vincent S.J., Chen F.K., Collins M.J. (2020). Effect of Altered OCT Image Quality on Deep Learning Boundary Segmentation. IEEE Access.

[29] Olsson H., Millward J.M., Starke L., Gladytz T., Klein T., Fehr J., Lai W.C., Lippert C., Niendorf T., Waiczies S. (2024). Simulating rigid head motion artifacts on brain magnitude MRI data–Outcome on image quality and segmentation of the cerebral cortex. PLoS ONE.

[30] Muthusivarajan R., Celaya A., Yung J.P., Long J.P., Viswanath S.E., Marcus D.S., Chung C., Fuentes D. (2024). Evaluating the relationship between magnetic resonance image quality metrics and deep learning-based segmentation accuracy of brain tumors. Med. Phys..

[31] Orlando N., Gyacskov I., Gillies D.J., Guo F., Romagnoli C., D’Souza D., Cool D.W., Hoover D.A., Fenster A. (2022). Effect of dataset size, image quality, and image type on deep learning-based automatic prostate segmentation in 3D ultrasound. Phys. Med. Biol..

[32] Marcinkiewicz M., Mrukwa G. (2019). Quantitative impact of label noise on the quality of segmentation of brain tumors on MRI scans. 2019 Federated Conference on Computer Science and Information Systems (FedCSIS).

[33] Nabors L.B., Portnow J., Ammirati M., Baehring J., Brem H., Butowski N., Fenstermaker R.A., Forsyth P., Hattangadi-Gluth J., Holdhoff M. (2017). Central nervous system cancers, version 1.2017 featured updates to the NCCN guidelines. JNCCN J. Natl. Compr. Cancer Netw..

[34] Stupp R., Brada M., van den Bent M.J., Tonn J.C., Pentheroudakis G. (2014). High-grade glioma: ESMO clinical practice guidelines for diagnosis, treatment and follow-up. Ann. Oncol..

[35] Stummer W., Pichlmeier U., Meinel T., Wiestler D., Zanella F., Reulen H.J. (2006). Fluorescence-guided surgery with 5-aminolevulinic acid for resection of malignant glioma: A randomised controlled multicentre phase III trial. Lancet Oncol..

[36] Sadri A.R., Janowczyk A., Zhou R., Verma R., Beig N., Antunes J., Madabhushi A., Tiwari P., Viswanath S.E. (2020). Technical Note: MRQy—An open-source tool for quality control of MR imaging data. Med. Phys..

[37] Holden Helland R., Bouget D., Eijgelaar R.S., De Witt Hamer P.C., Barkhof F., Solheim O., Reinertsen I., Linguraru M.G., Dou Q., Feragen A., Giannarou S., Glocker B., Lekadir K., Schnabel J.A. (2024). Glioblastoma Segmentation from Early Post-operative MRI: Challenges and Clinical Impact. International Conference on Medical Image Computing and Computer-Assisted Intervention.

[38] Oktay O., Schlemper J., Folgoc L.L., Lee M., Heinrich M., Misawa K., Mori K., McDonagh S., Hammerla N.Y., Kainz B. (2018). Attention U-Net: Learning Where to Look for the Pancreas. arXiv.

[39] Avants B.B., Tustison N., Song G. (2009). Advanced normalization tools (ANTS). Insight J..

[40] Cohen J. (2013). Statistical Power Analysis for the Behavioral Sciences.

[41] Sawilowsky S.S. (2009). New effect size rules of thumb. J. Mod. Appl. Stat. Methods.

[42] McMahan H.B., Moore E., Ramage D., Hampson S., y Arcas B.A. (2017). Communication-Efficient Learning of Deep Networks from Decentralized Data. Artificial Intelligence and Statistics.

[43] Zhang C., Xie Y., Bai H., Yu B., Li W., Gao Y. (2021). A survey on federated learning. Knowl.-Based Syst..

