# Supplementary material for: Assessing Impact of Data Quality in Early Post-Operative Glioblastoma Segmentation
Source: J Imaging. 2026 Feb 10;12(2):73. doi: 10.3390/jimaging12020073 (PMC12941527; doi:10.3390/jimaging12020073)
Supplement: Supplementary file 1 [file jimaging-12-00073-s001.zip › jimaging-4069467-supplementary.pdf]

Supplementary Material

1 Quality evaluation

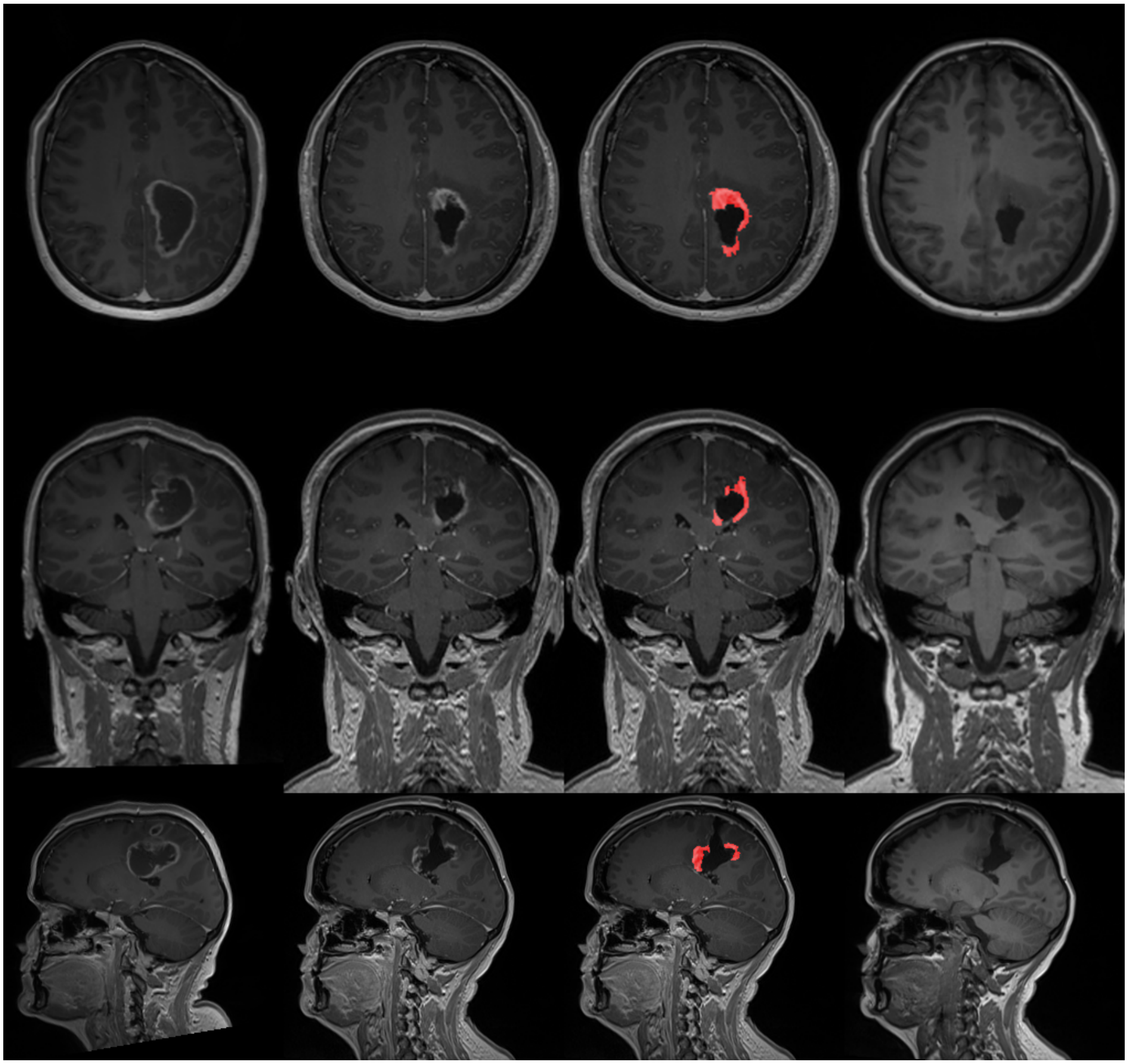

Figure S1: Example slide shown to a clinician for evaluation of image and annotation quality for one patient. From the left: pre-operative T1-CE, early post-operative T1-CE, early post-operative T1-CE with ground truth annotation, early post-operative T1w. From the top: axial, coronal and sagittal view.

2 Quality metrics

Table S1: Minimum, mean, and maximum slice thickness (ST) for each quality category.

**Quality group**	Count	Min ST (mm)	Mean ST (mm)	Max ST (mm)
Image Quality = 1	241	0.499	0.955	4.40
Image Quality = 2	14	1.0	4.125	6.0
Image Quality = 3	169	1.0	5.394	6.0
Annotation Quality = 1	289	0.499	2.88	6.0
Annotation Quality = 2	125	0.499	2.619	6.0
Annotation Quality = 3	10	1.0	3.8	6.0

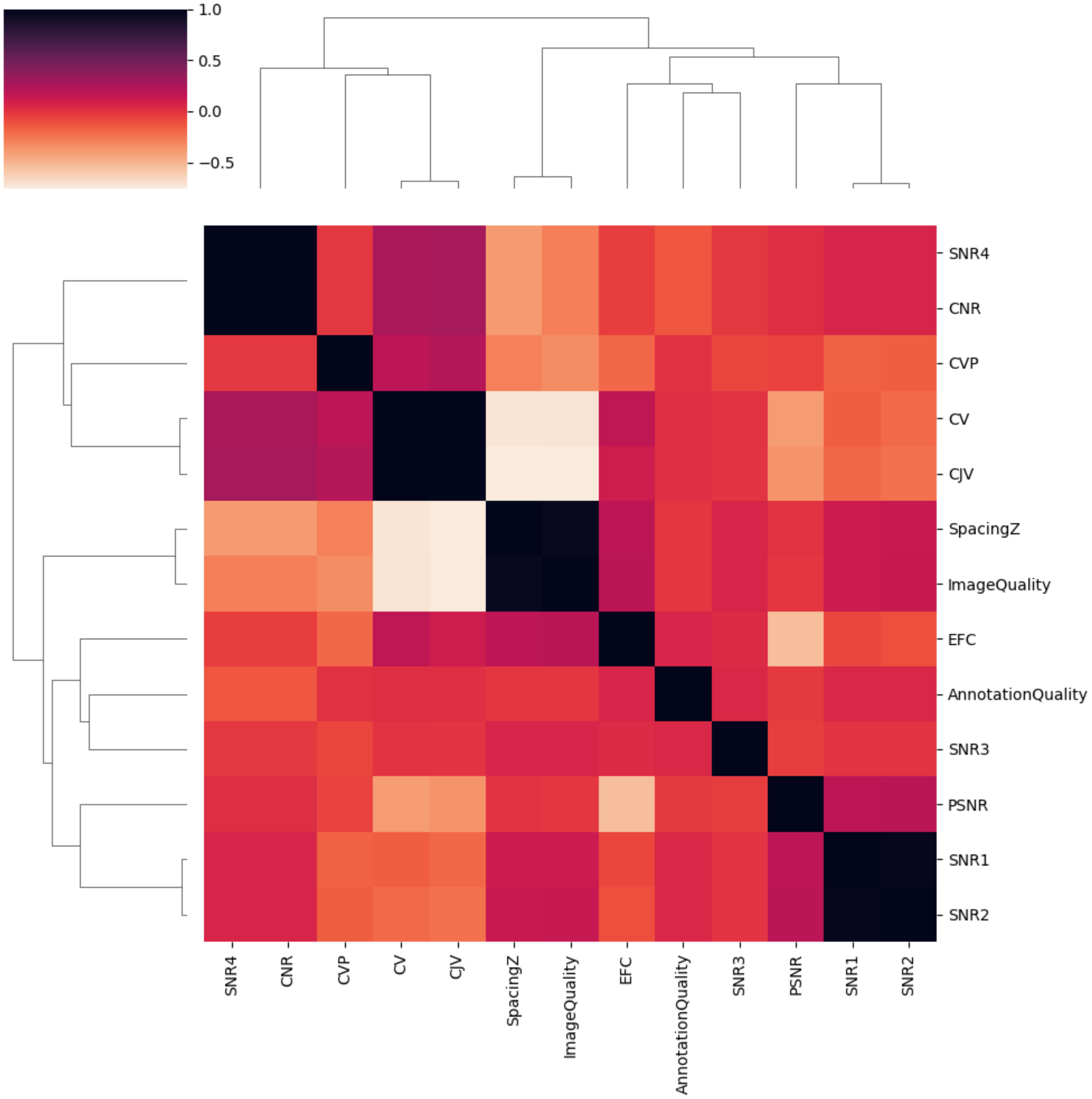

Figure S2: correlation coefficients between the evaluated qualities, slice thickness, and IQMs from MRQy .

To assess the validity of the quality evaluation metrics used in the study, quantitative image quality metrics (IQMs) were computed using MRQy[1]. These were compared against the evaluated image and annotation qualities using correlation

coefficients, in addition to slice thickness, as this was one of the main criteria for assessing image quality. The correlation coefficients are shown in Figure S 2. The evaluated qualities in our study are named ImageQuality and AnnotationQuality, respectively. The slice thickness is named SpacingZ, and the remaining variables are the selected IQMs, identical to the ones used in [2]. These are described in detail in [2], Table 1. The references for higher and lower image quality depend on the IQM. To improve the interpretability, some of the IQM values have been inverted, so that each of the metrics are arranged in the same order. Lower values signify high quality for all metrics, and higher values signify low quality.

3 Experiments

Table S2: Number of images available for training, number of positive images and positive ratio, and patients from each hospital, for each experiment

Experiment	N (train)	N Pos.	Pos. ratio	STO	SUH
1. ImAnAll	423	274	0.648	236	187
2. ImHigh	240	155	0.646	226	14
3. AnHigh	289	179	0.619	155	134
4. ImAnHigh	161	102	0.634	151	10

Table S3: Additional metrics: Mean absolute volume difference ± std between prediction and ground truth in ml (VD), Sum of predicted positives (PP), true positives (TP), false positives (FP), true negatives (TN), and false negatives (FN) over 5 folds per experiment.

Experiment	VD (ml)	PP	TP	FP	TN	FN
1. ImAnAll	1.08±2.46	370	260	110	39	14
2. ImHigh	1.60±2.77	380	252	128	21	22
3. AnHigh	1.21±2.69	378	265	113	36	9
4. ImAnHigh	1.48±3.00	371	249	122	27	25

References

1. Sadri, A. R. *et al.* Technical Note: MRQy - An open-source tool for quality control of MR imaging data. *Med. physics* **47**, 6029–6038, DOI: 10.1002/mp.14593 (2020).

2. Muthusivarajan, R. *et al.* Evaluating the relationship between magnetic resonance image quality metrics and deep learning-based segmentation accuracy of brain tumors, DOI: 10.1002/mp.17059 (2024).
